# Variable rather than extreme slow reaction times distinguish brain states during sustained attention

**DOI:** 10.1038/s41598-021-94161-0

**Published:** 2021-07-21

**Authors:** Ayumu Yamashita, David Rothlein, Aaron Kucyi, Eve M. Valera, Laura Germine, Jeremy Wilmer, Joseph DeGutis, Michael Esterman

**Affiliations:** 1 Department of Psychiatry, Boston University School of Medicine, Boston, MA 02118 USA; 2 Boston Attention and Learning Laboratory, VA Boston Healthcare System, Boston, MA 02130 USA; 3 Overseas Research Fellow, Japan Society for the Promotion of Science, Tokyo, 102-0083 Japan; 4 Department of Psychology, Northeastern University, Boston, MA 02115 USA; 5 Department of Psychiatry, Harvard Medical School, Massachusetts, 02215 USA; 6 Department of Psychiatry, Massachusetts General Hospital, Massachusetts, 02129 USA; 7 Institute for Technology in Psychiatry , McLean Hospital , Boston, MA 02478 USA; 8 Wellesley College, Wellesley, MA 02481 USA; 9 National Center for PTSD, VA Boston Healthcare System, Boston, MA 02130 USA

**Keywords:** Attention, Human behaviour

## Abstract

A common behavioral marker of optimal attention focus is faster responses or reduced response variability. Our previous study found two dominant brain states during sustained attention, and these states differed in their behavioral accuracy and reaction time (RT) variability. However, RT distributions are often positively skewed with a long tail (i.e., reflecting occasional slow responses). Therefore, a larger RT variance could also be explained by this long tail rather than the variance around an assumed normal distribution (i.e., reflecting pervasive response instability based on both faster and slower responses). Resolving this ambiguity is important for better understanding mechanisms of sustained attention. Here, using a large dataset of over 20,000 participants who performed a sustained attention task, we first demonstrated the utility of the exGuassian distribution that can decompose RTs into a strategy factor, a variance factor, and a long tail factor. We then investigated which factor(s) differed between the two brain states using fMRI. Across two independent datasets, results indicate unambiguously that the variance factor differs between the two dominant brain states. These findings indicate that ‘suboptimal’ is different from ‘slow’ at the behavior and neural level, and have implications for theoretically and methodologically guiding future sustained attention research.

## Introduction

Although sustaining attention over time is important for our everyday life, evidence suggests our attention fluctuates from moment to moment despite our efforts to maintain optimal focus. Previous studies have found significant individual differences in sustained attention ability in the healthy population, and have related these differences to real world outcomes such as driving accidents, school performance, and memory encoding^1–5^. Furthermore, impairments in sustained attention are common in a diverse range of clinical populations such as attention-deficit/hyperactivity disorder (ADHD)^6–8^, major depressive disorder^9^, schizophrenia^10^, bipolar disorder^11^, post-traumatic stress disorder^12–15^, early-life trauma^16^, and traumatic brain injury^17^. Therefore, revealing the neural and cognitive mechanisms required to sustain optimal attention is an important issue in modern society.

To better characterize sustained attention, attentional states have been behaviorally estimated using response accuracy^17–19^ and reaction time (RT) to stimuli^6,20–23^. In particular, RT is frequently used in a variety of cognitive tasks regardless of the difficulty of the cognitive tasks. For example, previous studies found that fast RTs frequently occur before commission errors, thought to reflect lapses due to impulsiveness or mindlessness. This interpretation is also consistent with the result which shows that fast RTs are associated with task-unrelated thoughts^17,20,23^. On the other hand, slow RTs frequently occur before omission errors, thought to reflect more severe attentional lapses due to task disengagement^20^. Therefore, both fast RTs, which are interpreted as impulsivity and mindless automatic responding, and slow RTs, which are interpreted as disengaged/inefficient processing of stimulus information, are related to inattention. As an alternative to the speed of RT, intra-individual variability of RT has been recognized as an important indicator of sustained attention^21,24,25^. Attention fluctuates between stable/accurate (in-the-zone) and variable/error-prone (out-of-the-zone) states^21,26^. Furthermore, one of the most consistent manifestations of ADHD is the high prevalence of intra-individual RT variability^6,8^.

**Figure 1:**
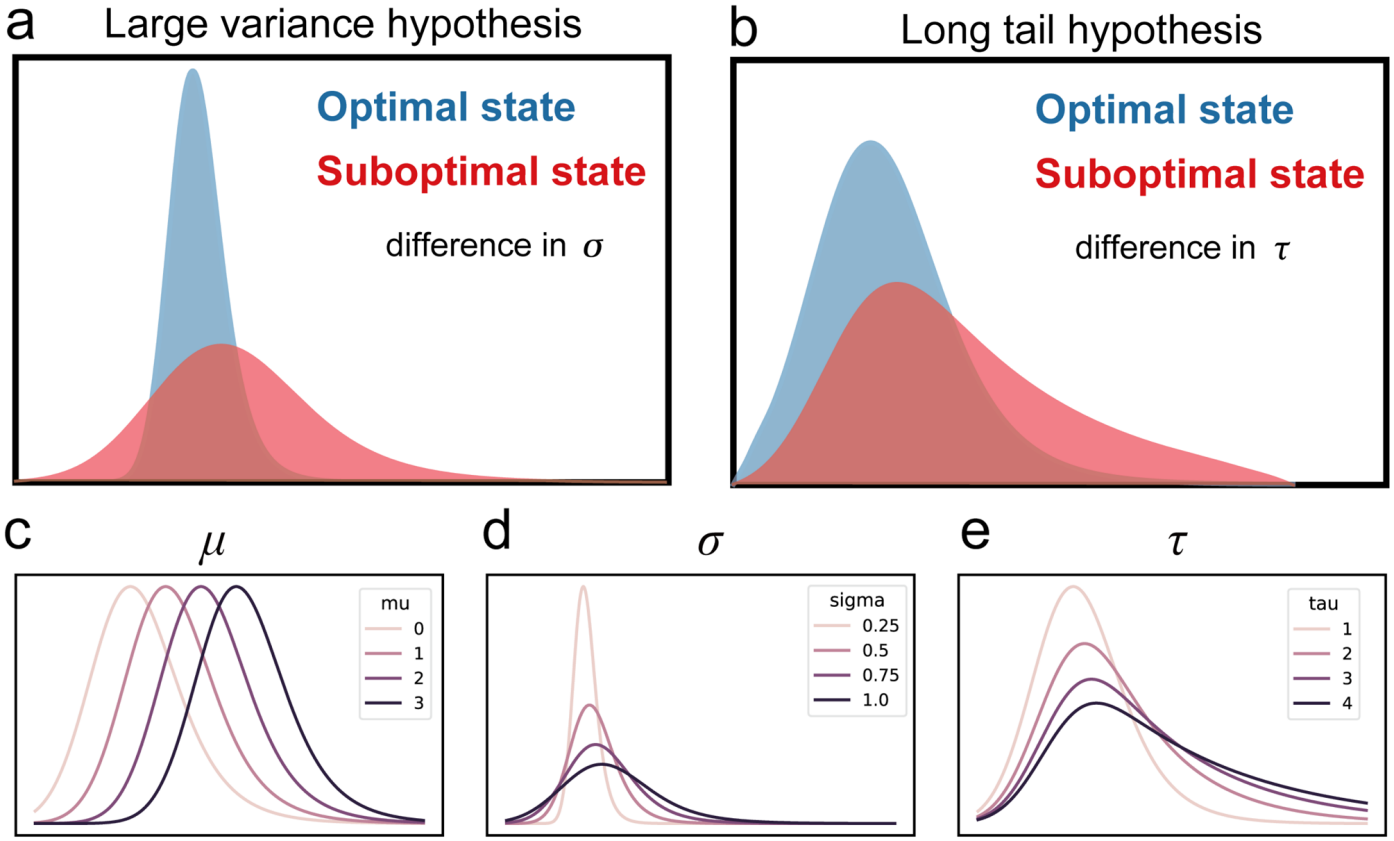
Two alternative hypotheses and exGaussian distributions. (**a**) Illustration of large variance hypothesis. (**b**) Illustration of long tail hypothesis. (**c**) exGaussian distributions with different *μ* (*μ* = {0, 1, 2, 3}, *σ* = 1, *τ* = 1). (**d**) exGaussian distributions with different *σ* (*μ* = 0, *σ* = {0.25, 0.50, 0.75, 1.0}, *τ* = 1). (**e**) exGaussian distributions with different *τ* (*μ* = 0, *σ* = 1, *τ* = {1, 2, 3, 4}).

From the viewpoint of the RT distribution across task trials, the mean of the RTs represents the response speed while the variance of the RTs represents the intra-individual variability. Sustained attention tasks vary considerably. They include simple reaction time tasks such as the psychomotor vigilance task (PVT), which do not have a response deadline, and continuous performance tasks (CPT) such as sustained attention to response task (SART) and gradual onset CPT (gradCPT), which have an implicit deadline for a response as the trials follow in rapid succession. Given this implicit response deadline in CPTs, which does not allow extremely slow responses, mean and variance had been the main focus of research using these tools. While RT mean and variance are intuitive and useful, these parameters are usually inappropriate because, despite response deadlines, RT distributions rarely conform to Gaussian distribution. RT distributions are typically positively skewed with a long tail indicating more extreme slow responses than would be found in a Gaussian distribution. That is, a large RT variance could be fully accounted for by the long tail factor of the RT distribution. Hence, we believe that distinguishing the distributional drivers of RT variability is important to better tease apart the information contained in RT distributions and their relationship to sustained attention. The exGaussian distribution is commonly used to decompose RTs into *μ* and *σ*, that, respectively, describe the mean and standard deviation (variability) of the Gaussian distribution, and an independent exponentially distributed variable, *τ*, that accounts for the positive skew (Fig. 1c–e)^27,28^. Theoretically, *mean RT* = *μ* + *τ*, and *RT variance* = *σ*^2^ + *τ*^*2*^. Among the three ex-Gaussian parameters, a previous study of sustained attention in ADHD in adolescents showed *τ* was specifically positively associated with omission errors, suggesting more frequent disengagement-based attention lapses; *μ* was significantly negatively associated with commission errors suggesting an impulsive, or mindless response style^29^. Further, this study and others found that ADHD was not associated with generalized RT slowing, which is characterized by *μ*, but rather was associated with both the long tail factor of slower RT, which is characterized by *τ,* and with the variance factor, which is characterized by *σ*^29,30^. However, there are still only a few studies applying exGaussian distribution to RTs in sustained attention tasks such as continuous performance task^22,29–31^. Therefore, in the first part of this study, we examined the relationship between exGaussian parameters and sustained attention performance using a big dataset including over 20,000 participants.

In the second part of this study, we used these tools to investigate the neural mechanism of sustained attention. In our previous study, we observed two dominant brain states during sustained attention task using functional magnetic resonance imaging (fMRI)^32^. One state (State1) was characterized by activation of frontoparietal network subsystem A (FPN_A_), default mode network (DMN) and limbic network, and the other state (State2) was characterized as activation of frontoparietal network subsystem B (FPN_B_), dorsal attention network (DAN), salience network (SN), somato-motor (SMN) and visual network. RT variance was lower and accuracy was higher during State1 than during State2, suggesting State1 was optimal and State2 was suboptimal. However, since this study assumed a Gaussian RT distribution, it is not known whether the difference in RT variance between brain states was due to the variance factor or the long tail factor. Determining which factor(s) differ between brain states is important for better understanding the mechanisms of sustained attention. For example, differences in the variance factor, on the one hand, suggests attentional/brain states reflects variability across the entire empirical RT distribution, including both impulsive fast RT and slow RTs (Large variance hypothesis; Fig. 1a). Previous studies suggest this factor may represent exploration in the environment to gather information or adjust cognitive control settings^33^. On the other hand, differences in the long tail factor suggests attentional/brain states reflects only more infrequent and extremely slow RTs suggesting occasional attention lapses (Long tail hypothesis; Fig. 1b)^22,29,34^. Here, by comparing exGaussian parameters between these two brain states, we can help resolve this ambiguity present in many studies, including our recent work. For the purpose of replication, we tested each of these two hypotheses using two different independent fMRI datasets.

## Materials and methods

### Overview

We conducted our analyses with three datasets. In all datasets, all participants performed a gradCPT. The gradCPT is a well-validated test of sustained attention, previously used to define attentional states by reaction time variability fluctuations over time^2,21,26^. Dataset1 consists of gradCPT behavioral data from 21,406 participants between 10 and 70 years old who performed one session of 4 min gradCPT with an interstimulus interval (ISI) of 800 ms obtained via the website testmybrain.org^2^. Dataset1 was used to investigate the relationship between exGaussian parameters and sustained attention performance. Dataset2 consists of gradCPT behavioral data with fMRI from 16 participants who performed three runs of 8 min gradCPT with ISI of 800 ms in MRI scanner^21^. Dataset2 was used to investigate the difference in exGaussian parameters between brain states. Dataset3 consists of gradCPT behavioral data with fMRI from 29 participants who performed four runs with thought probes of 9 min gradCPT with ISI of 1300 ms in MRI scanner^35^. Dataset3 was used to replicate the results in the Dataset2.

### Behavioral analysis

#### gradual onset continuous performance task

The gradCPT contained 10 round, grayscale photographs of mountain scenes and 10 of city scenes. These scenes were randomly presented with 10% mountain and 90% city trials, without allowing the identical scene to repeat on consecutive trials. Scene images gradually transitioned from one to the next, using a linear pixel-by-pixel interpolation, with each transition occurring in 800 ms (fast version; Dataset1 and Dataset2) or 1300 ms (slow version; Dataset3). Participants were instructed to press a button for each city scene, and withhold responses to mountain scenes. Response accuracy was emphasized without reference to speed. However, given that the next stimulus would replace the current stimulus in 800/1300 ms, a response deadline was implicit in the task. In the Dataset1, the order of presented images was the same for all participants, but in the Dataset2 and 3, the order of presented images was randomized individually. This method is the same as the one used in our previous studies^21,32^.

#### Calculation of reaction time

RT was calculated relative to the beginning of each image transition, such that an RT of 800/1300 ms (slow and fast versions) indicates a button press at the moment image n was 100% coherent and not mixed with other images. A shorter RT indicates that the current scene was still in the process of transitioning from the previous, and a longer RT indicates that the current scene was in the process of transitioning to the subsequent scene. So, for example, an RT of 720/1170 ms would be at the moment of 90% image n and 10% image n − 1, and so forth. On rare trials with highly deviant RTs (before 70% coherence of image n and after 40% coherence of image n + 1) or multiple button presses, an iterative algorithm maximized correct responses as follows. The algorithm first assigned unambiguous correct responses, leaving few ambiguous button presses (presses before 70% coherence of the current scene and after 40% coherence of the following scene or multiple presses occurred on < 5% of trials). Second, ambiguous presses were assigned to an adjacent trial if 1 of the 2 had no response. If both adjacent trials had no response, the press was assigned to the closest trial, unless one was a no-go target, in which case subjects were given the benefit of the doubt that they correctly omitted. Finally, if there were multiple presses that could be assigned to any 1 trial, the fastest response was selected. Slight variations to this algorithm yielded highly similar results, as most button presses showed a 1–1 correspondence with presented images^21^.

#### Estimation of exGaussian distribution parameters

We estimated exGaussian parameters of RTs (*μ*, *σ*, *τ*) using only correct commission responses (press to city image) for every participant. To estimate parameters, we used maximum likelihood estimation method implemented in ScyiPy version 1.5.2^36^. As a comparison, we further estimated the parameters of the Gaussian distribution (*μ*_*normal*_, *σ*_*normal*_) in the same way. We compared which distribution fit the data better by comparing the *R*-squared values between the actual data and the simulated data obtained using the estimated parameters. Note, we found almost half of participants had negative skewness in their RT distribution, which indicates the long tail for fast RT, in Dataset1 (11,291 of 21,406 participants). Since the assumed exGaussian distribution is not appropriate for negative skewed distributions, we estimated exGaussian parameters (*μ*, *σ*, *τ*) using flipped RT distribution (reverse the positive and negative values of RT) for participants with negative skewness. In this way, we were able to improve the goodness of fit for participants with negative skewness (Supplementary Fig. S1). When we integrate the parameters with the participants with positive skewness, we flipped the value of *μ,* but used *σ* and *τ* to put them on the same scale. Therefore, *τ* was initially interpreted as the long tail of the RT distribution regardless of which direction in the Dataset1. We note that interpretation of the negative skewness is difficult, as a very slow RT is possibly regarded as a fast RT in the next trial due to the nature of the gradCPT. To support this idea, we found significant difference in mean RT between individuals with positive skewness versus negative skewness (Positive skewness: mean RT = 850 ms; Negative skewness: mean RT = 888 ms, *t*_*21404*_ = 40.9, *p* < 0.001, Hedges' *g* = 0.56, two sample *t*-test). From this result, we can speculate that a very slow RT from individuals with slower RTs on average is likely to be regarded as a fast RT on the next trial (and omission error to current trial). This may explain why individuals with negative skewness had slower mean RT. However, a previous study shows the existence of a type of error called anticipation that indicates pressing a button in anticipation of a stimulus resulting in a very fast RT^20^. Thus, it is difficult to interpret whether the negative skewness really represents frequent fast RTs in the Dataset1, or rather slow RTs to the previous stimulus. Given this ambiguity, we also conducted the analyses of Dataset1 separately for both positive and negative skewed participants, which revealed mostly consistent results, albeit with some differences. Thus, to avoid this ambiguity, for Dataset2 and Dataset3, as most of participants had positive skewness (81% and 86%, respectively), participants with negative skewness were excluded from the analysis so that the long tail factor can be interpreted as slow RTs unambiguously. We found mean RTs were significantly slower in Dataset1 than those in the Dataset2, which had the same ISI (Dataset1: mean RT = 871 ms; Dataset2: mean RT = 744 ms, *t*_*21420*_ = 7.49, *p* < 0.001, Hedges' *g* = 1.89, two sample *t*-test). From this result, we can speculate that this is the reason why there were many participants with negative skewness in Dataset1 with the same logic as above, which could be driven by technical differences in response collection (web vs. lab)^2^. Note that when we included participants with negative skewness in the analysis by estimating exGaussian parameters using flipped RT method described above, the pattern of the results did not change in Dataset2 and Dataset3 (Supplementary Fig. S2).

#### Calculation of accuracy

In addition to RT related performance, we also calculated accuracy of response to investigate the relationship with exGaussian parameters in Dataset1. Trials in which participants erroneously inhibited a button press to city images were considered omission errors. Trials in which participants erroneously responded to mountain images were considered commission errors. We calculated the Pearson correlation coefficient between exGaussian parameters (*μ*, *σ*, *τ*) and the rate of omission and commission errors, respectively.

### fMRI analysis

The following descriptions were exactly the same methodology as in our previous study^32^.

#### Preprocessing

A preprocessing of the fMRI data was performed using FMRIPREP version 1.3.0^37^. Preprocessing steps were realignment, coregistration, segmentation of T1-weighted structural images, normalization to Montreal Neurological Institute space. For more details of the pipeline, see http://fmriprep.readthedocs.io/en/latest/workflows.html.

#### fMRI signal extraction from brain networks

We used the 400 regions of interest (ROIs) that were defined as 4-mm spheres around the center coordinates^38^. The blood oxygen level dependent (BOLD) signal time courses were extracted with spatial smoothing using an isotropic Gaussian kernel of 6 mm full-width at half-maximum from these 400 ROIs.

We removed several sources of spurious variance from 400 ROIs’ signal time courses by using linear regression with eighteen regression parameters, including six motion parameters, an average signal over the whole brain, five event-related task regressors on the BOLD response time course and six physiological noise regressors. To account for task events (commission error, correct omission, correct commission, and omission error) and trial-to-trial RT, we estimated five BOLD response time courses of each event type by using *hemodynamic_models* function implemented in Nistat (https://nistats.github.io/). To account for the physiological confounding, we extracted six physiological noise regressors by applying anatomical CompCor (aCompCor)^39^. We obtained a mask to exclude signals with a cortical origin by eroding the brain mask and ensuring that it contained subcortical structures only. Six aCompCor components were calculated within the intersection of the subcortical mask and union of the CSF and WM masks calculated in T1-weighted image space after their projection to the native space of functional images in each session. Finally, to account for variance and mean differences across run and participant, we standardized the BOLD signal time course for each ROI (shifted to zero mean and scaled to unit variance) after noise regression. These ROIs were then classified into seven functionally different brain networks that were determined in the previous studies^38^. A recent study identified two distinct subsystems within the FPN. FPN_A_ exhibited stronger connectivity with the DMN than the DAN, whereas FPN_B_ exhibited the opposite pattern^40^. Therefore, we split the FPN into FPN_A_ and FPN_B_ using Yeo’s 17 network^38^ and classified into eight functionally different brain networks in the end. For each participant, we then calculated eight time series that represented the activity of these brain networks by averaging BOLD signal time courses in the 400 ROIs corresponding to those brain networks. Note, using the voxel-wise network average time series instead of the ROI-wise network average produced nearly identical patterns of results.

#### Pairwise maximum entropy model

The pairwise Maximum entropy model (MEM) was applied to the preprocessed BOLD signals as follows in the same manner as that employed in previous studies^41–46^. To conduct this analysis, we used open Energy Landscape Analysis Toolkit (https://sites.google.com/site/ezakitakahiro/software). For each network activity time series, the obtained BOLD signals were binarized with a threshold that was defined as the time-averaged activity of the network activity. We then concatenated BOLD signals from all runs and all participants for each network activity. In this method, the binarized activity ${\sigma }_{i}^{t}$ at network *i* and discrete time *t* represents either active or inactive (+ 1 or 0). The activity pattern at time *t* is described by ${V}^{t}= {\left[{\sigma }_{1}^{t}, {\sigma }_{2}^{t}, \dots , {\sigma }_{N}^{t}\right]}^{\mathrm{T}}$ where *N* is the number of the networks. In our case, the number of the networks was eight. The *k* th brain activity pattern is described by ${V}_{k}$ $\left(k=1, 2, \dots , {2}^{N}\right)$. The probability distribution of the *k* th brain activity pattern (${V}_{k}$) with the largest entropy follows the Boltzmann distribution, when the empirical activation of network *i*, $\langle {\sigma }_{i}\rangle =\left(1/T\right){\sum }_{t=1}^{T}{\sigma }_{i}^{t}$, and the empirical pairwise interaction between networks *i* and *j*, $\langle {\sigma }_{i}{\sigma }_{j}\rangle =\left(1/T\right){\sum }_{t=1}^{T}{\sigma }_{i}^{t}{\sigma }_{j}^{t}$ are estimated from the data, where *T* is the number of volumes^47^. That is, $P\left({V}_{k}\right)={e}^{E\left({V}_{k}\right)}/{\sum }_{l=1}^{{2}^{N}}{e}^{-E\left({V}_{l}\right)}$, where $E\left({V}_{k}\right)$ is the energy of activity pattern ${V}_{k}$ and is given by

1 $$E\left({V}_{k}\right)=-{\sum }_{i=1}^{N}{h}_{i}{\sigma }_{i}\left({V}_{k}\right)-\frac{1}{2}{\sum }_{i=1}^{N}{\sum }_{j=1, j\ne i}^{N}{J}_{ij}{\sigma }_{i}\left({V}_{k}\right){\sigma }_{j}\left({V}_{k}\right)$$

,${\sigma }_{i}\left({V}_{k}\right)$ is the binarized activity (+ 1 or 0) at network *i* under activity pattern ${V}_{k}$. We estimated ${h}_{i}$ and ${J}_{ij}$ by maximum likelihood estimation to adjust the model-based activation of network *i*,${\langle {\sigma }_{i}\rangle }_{model} (={h}_{i}$) and the model-based pairwise interaction between networks *i* and *j*,${\langle {\sigma }_{i}{\sigma }_{j}\rangle }_{model}$ $(={J}_{ij}$) toward the empirical activation $\langle {\sigma }_{i}\rangle$ and the empirical pairwise interaction $\langle {\sigma }_{i}{\sigma }_{j}\rangle$,${\langle {\sigma }_{i}\rangle }_{model}={\sum }_{l=1}^{{2}^{N}}{\sigma }_{i}\left({V}_{l}\right)P\left({V}_{l}\right)$ and ${\langle {\sigma }_{i}{\sigma }_{j}\rangle }_{model}={\sum }_{l=1}^{{2}^{N}}{\sigma }_{i}\left({V}_{l}\right){\sigma }_{j}\left({V}_{l}\right)P\left({V}_{l}\right)$. The likelihood was maximized by a gradient ascent scheme. Please see the previous study for more details how to estimate ${h}_{i}$ and ${J}_{ij}$^42^,

We confirmed whether the pairwise MEM accurately fit to the data by calculating Pearson’s correlation coefficient between empirical appearance probability and model appearance probability $P\left({V}_{k}\right)$. Empirical appearance probability of brain activity pattern ${V}_{k}$ is calculated by $\left(1/T\right){\sum }_{t=1}^{T}{z}_{k}^{t}$, where ${\mathbf{z}}^{t}$ is a *K*-dimensional binary variable having a 1-of-*K* representation in which a particular element ${z}_{k}^{t}$ is equal to 1 and all other elements are equal to 0. The values of ${z}_{k}^{t}$ therefore satisfy ${z}_{k}^{t} \in \left\{\mathrm{0,1}\right\}$ and $\sum_{k}{z}_{k}^{t}=1$, and we see that there are *K* ($={2}^{N}$) possible brain activity patterns for the vector ${\mathbf{z}}^{t}$ at time *t* according to which element is nonzero. In non-technical terms, this confirms whether the model successfully fit the data.

#### Energy landscape analysis (Definition of brain state)

The energy landscape is defined as a graph of brain activity patterns ${V}_{k}$ with the corresponding energy $E\left({V}_{k}\right)$ as done in the previous studies^41,42,45,46^. We first exhaustively searched for local energy minima that has the smallest energy value among those of all the *N* adjacent patterns. Two brain activity patterns are regarded as adjacent if they take the opposite binary activity at just one brain region. Thus, we can discover some local minimum brain activity patterns. Therefore, the number of brain states (local minimum brain states) is determined in a data-driven manner. We then summarized all brain activity patterns into local minimum brain states in the following manner. We first selected a starting brain activity pattern *k* among the ${2}^{N}$ brain activity patterns. Then, if any of its neighbor patterns has a smaller value of energy than pattern *k*, we moved to the neighbor pattern with the smallest energy value. Otherwise, we did not move, which implied that pattern *k* was a local minimum. We repeated this procedure until arriving at a local minimum. The starting pattern *k* was regarded to belong to the local minimum brain state that was finally reached. We estimated the corresponding local minimum brain state for all brain activity patterns, so that we could estimate the time series of brain states from the time series of brain activity patterns.

#### exGaussian parameters in each brain state

We observed two dominant brain states during the sustained attention task using the energy landscape analysis^32^. State1 was characterized by activation of FPN_A_, DMN and limbic network, and State2 was characterized as activation of FPN_B_, DAN, SAN, SMN and visual network. After defining these brain states, all participants had a brain state transition time series and a RT time series. Thus, RTs were gathered for each brain state, and exGaussian parameters were estimated using the RTs for each brain state in each participant. To account for the hemodynamic response lag, we shifted the time labels of the brain states backwards by 5 s. To statistically investigate the difference in parameters between brain states, we conducted two-tailed paired *t*-tests and calculated Hedges’ *g* as the effect size^48,49^. We did not control for multiple comparisons for this analysis, because we confirmed its reproducibility by performing the same analysis on two independent datasets.

### Details in each dataset

#### Dataset1

We included 21,406 volunteers between the ages of 10 and 70 years (mean = 30.48, sd = 13.27) in the analyses. Participants were visitors to TestMyBrain.org, a cognitive testing web site where members of the public participants in research studies in exchange for individualized feedback about their performance^2,50,51^. Data from repeat participation were excluded. Overall, 25,274 participants completed the task, and 21,406 passed quality control measures (missing data or technical problems)^2^. Among technical problems, we chose to exclude testing sessions that exceeded 10% error in the average stimulus presentation time, that is, the time it took for a new image to transition from 0 to 100% opacity. Other participants were excluded for “tune-outs,” defined as intervals of 30 s or more without a response. Of the 21,406 participants that were included, there was a nearly equal ratio of males and females (11,540 males, 9827 females, 39 unknown). The study and all analyses were approved by the Committee for the Use of Human Subjects at Harvard University, and written informed consent was obtained from all participants. It was performed in accordance to the relevant guidelines and conforms to the principles of the Declaration of Helsinki. The data used in this study and portions of the methods have been published^2,51^, but the current analyses and results have not been published elsewhere.

#### Dataset2

Sixteen participants (6 males, ages 18–34 years, mean age = 24.1 years) performed the gradCPT during fMRI. Subjects completed the three 8-min gradCPT runs. All participants were right-handed, with normal or corrected-to-normal vision and no reported history of major medical illness, head trauma, neurological, or psychiatric disorder. The study and all analyses were approved by the VA Boston Healthcare System IRB, and written informed consent was obtained from all participants. It was performed in accordance to the relevant guidelines and conforms to the principles of the Declaration of Helsinki. The data used in this study and portions of the methods have been previously published^21,32^, but the current analyses and results have not been published elsewhere.

Scanning was performed on a 3 T Siemens MAGNETOM Trio system equipped with a 12-channel head coil, at the VA Boston Neuroimaging Research Center. Functional runs included 248 whole-brain volumes acquired using an echo-planar imaging sequence with the following parameters: repetition time (TR) = 2,000 ms, echo time (TE) = 30 ms, flip angle = 90°, acquisition matrix = 64 × 64, in-plane resolution = 3.0 mm^2^, 33 oblique slices, slice thickness = 3, 0.75 mm gap. MPRAGE parameters were as follows: TE = 3.32 ms, TR = 2530 ms, flip angle = 7°, acquisition matrix = 256 × 256, in-plane resolution = 1.0 mm^2^ slice thickness = 1.0 mm.

#### Dataset3

We attempted to replicate the results using independent Dataset 3. Twenty-nine participants (13 males, ages 21–36 years, mean age = 26.4 years) performed the long ISI gradCPT (1300 ms compared to 800 ms in the Dataset 2) during fMRI. Participants performed the gradCPT, modified here to include thought-probes to investigate mind wandering. Subjects completed the four long ISI gradCPT runs with intermittent thought-probes. Subjects were screened by phone and at an initial visit before the day of neuroimaging, where subjects were also trained on performing the long ISI gradCPT. Exclusion criteria were as follows: current mood, psychotic, or anxiety disorders (excluding simple phobias), ADHD, current use of psychotropic medication, full-scale IQ less than 80, neurological disorders, sensorimotor handicaps, current alcohol or substance abuse/dependence, and claustrophobia. The study and all analyses were approved by the Partners Human Research Institutional Review Board, and written consent was obtained from all participants. It was performed in accordance to the relevant guidelines and conforms to the principles of the Declaration of Helsinki. The data used in this study and portions of the methods have been published^32,52,53^, but the current analyses and results have not been published elsewhere.

Participants performed the gradCPT including thought-probes. Thought-probes appeared every 44–60 s pseudo-randomly (three possible durations of 44, 52, and 60 s). Upon the thought-probe, a question “To what degree was your focus just on the task or on something else?” was displayed. A continuous scale appeared with far-right and far-left anchors of “only task” and “only else”, respectively. Subjects pressed buttons with their middle and ring fingers to move the scale left and right, and with their thumb to enter their response. Responses were recorded on a graded scale of integers (not visible to the subjects) ranging from 0 (only task) to 100 (only else). The gradCPT immediately resumed after subjects entered their question responses (except for the last thought-probe in the run). This method is the same as the one used in our previous studies^32,52,53^.

Scanning was performed on a 3 T Siemens CONNECTOM scanner with a custom-made 64-channel phased array head coil, housed at the Athinoula A. Martinos Center for Biomedical Imaging. Functional runs included about 490 whole-brain volumes acquired using a multiband, echo-planar imaging (simultaneous multislice factor of 4) with the following parameters: TR = 1,080 ms, TE = 30 ms, flip angle = 60°, acquisition matrix = 55 × 55, in-plane resolution = 2.0 mm^2^, 68 oblique slices, slice thickness = 2. MPRAGE parameters were as follows: TE = 1.15 ms, TR = 2530 ms, flip angle = 7°, acquisition matrix = 256 × 256, in-plane resolution = 1.0 mm^2^ slice thickness = 1.0 mm.

## Results

### Relationship between sustained attention performance and distribution parameters in Dataset1

We first confirmed the exGaussian distribution (or reversed exGaussian) better fit the data than the Gaussian distribution (Positive skewness group, exGaussian: *R* squared = 0.980, Gaussian: *R* squared = 0.971, *t*_10114_ = 52.9, *p* < 0.001, Hedges’ *g* = 0.51; Negative skewness group, reversed exGaussian: *R* squared = 0.974, Gaussian: *R* squared = 0.962, *t*_11290_ = 47.1, *p* < 0.001, Hedges’ *g* = 0.43, paired *t*-test, Supplementary Fig. S1). Note, the goodness of fit (*R* squared) for Gaussian distribution was still high (*R* squared > 0.9). Descriptive statistics values of exGaussian parameters are summarized in Table 1. Given the relatively high skew and kurtosis, we report Spearman correlations (a common rule of thumb for normality is +/− 2 and 6, respectively, for skew and kurtosis^54^). Furthermore, we assessed the reliability of these parameters by using a split-half method. Namely, we divided into the first 2 min and the second 2 min and fitted exGaussian distribution (or reversed exGaussian) separately to each half, and calculated the Spearman-Brown corrected reliability. We found acceptable to high reliability for parameters (All participants, *μ*: reliability = 0.78; *σ*: reliability = 0.76; *τ*: reliability = 0.5). Note that although this version of the task is only 4 min in duration, we confirmed that there were significant performance decrements (accuracy decreased and RT variance increased) over time (Supplementary Fig. S3) indicating this abbreviated version of the task did tax sustained attention.

**Table 1:** Descriptive statistics values in exGaussian parameters.

Descriptive statistics values	Mean	Standard deviation	Skewness	Kurtosis
**Positive Skewness group**				
$\mu$	0.80	0.06	-0.17	1.02
$\sigma$	0.08	0.03	1.55	3.47
$\tau$	0.05	0.02	1.00	7.94
**Negative Skewness group**				
$\mu$	0.97	0.10	0.43	-0.13
$\sigma$	0.10	0.03	1.18	2.30
$\tau$	0.08	0.05	1.55	3.12
**All participants**				
$\mu$	0.89	0.12	0.51	-0.08
$\sigma$	0.09	0.03	1.21	2.27
$\tau$	0.07	0.04	2.04	6.14

**Figure 2:**
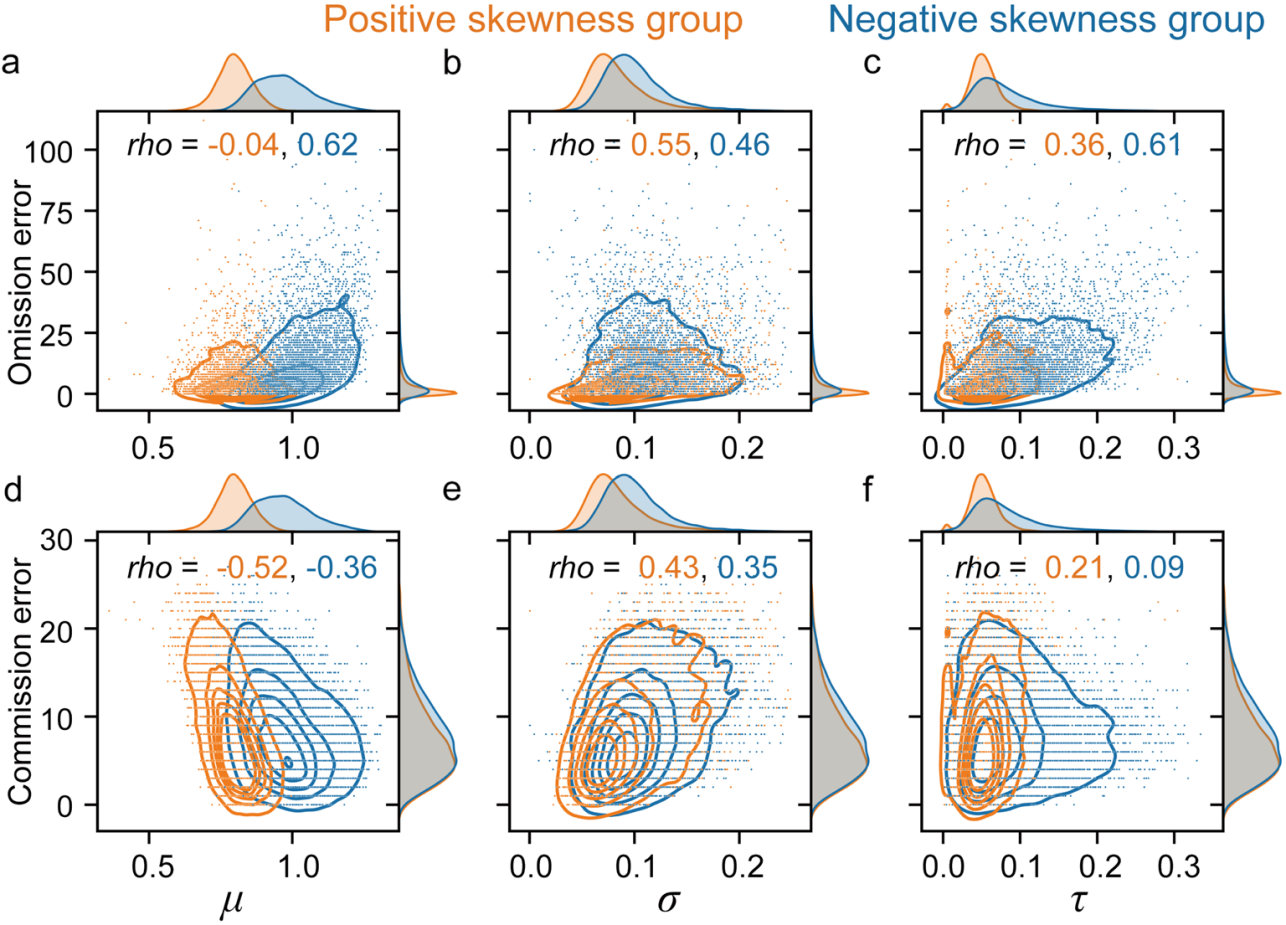
Relationship between exGaussian distribution parameters and sustained attention performance in each skewness group. (**a**) Scatter plot and histograms of *μ* and the number of omission errors. (**b**) Scatter plot and histograms of *σ* and the number of omission errors. (**c**) Scatter plot and histograms of *τ* and the number of omission errors. (**d**) Scatter plot and histograms of *μ* and the number of commission errors. (**e**) Scatter plot and histograms of of *σ* and the number of commission errors. (**f**) Scatter plot and histograms of *τ* and the number of commission errors. Solid line indicates a kernel density estimate, which is a method for visualizing the distribution of observations. Spearman’s correlation coefficients values were shown in each panel. Orange color indicates positive skewness group and blue color indicates negative skewness group.

Next, we investigated relationship between exGaussian parameters and errors by using all participants. We found that *μ* was moderately positively correlated with the number of omission errors and negatively correlated with the number of commission errors across individuals (Omission errors: Spearman’s *rho* = 0.43, *p* < 0.001, Supplementary Fig. S4a; Commission errors: Spearman’s *rho* = − 0.23, *p* < 0.001, Supplementary Fig. S4d). This result indicates that the participants who tend to respond slowly (larger *μ*) have more frequent omission errors and fewer commission errors, and vice versa. In other words, *μ* could represent an individual strategy factor, which is the same interpretation as average RT and criterion in our previous studies assuming a Gaussian distribution^2,55^. Next, we found *σ* was moderately positively correlated with both the number of omission errors and the number of commission errors (Omission error: Spearman’s *r* = 0.56, *p* < 0.001, Supplementary Fig. S4b; Commission error: Spearman’s *r* = 0.39, *p* < 0.001, Supplementary Fig. S4e), consistent with previous interpretation of RT variability as reflecting an ability factor^2^. On the other hand, we found *τ* was considerably positively correlated with the number of omission errors and weakly positively correlated with the number of commission errors (Omission errors: Spearman’s *r* = 0.55, *p* < 0.001, Supplementary Fig. S4c; Commission errors: Spearman’s *r* = 0.17, *p* < 0.001, Supplementary Fig. S4f). Furthermore, a multiple regression analysis with each error as the dependent variable, yielded significant coefficients for every parameter differently (see Table 2). We found that the number of omission errors was best explained by *τ* and the number of commission errors was best explained by *σ*. These results indicate that fitting exGaussian could separate the strategy factor (*μ*), the variability factor (*σ*), and the long tail factor (*τ*), because all parameters have different relationship with sustained attention performance. We next investigated the relationships in negative skewness group and positive skewness group separately. The patterns of the result were similar except the relationship between *μ* and the number of omission error was absent in positive skewness group (Fig. 2). Given these differences, we focused on the un-ambiguous positive skew group in the subsequent datasets, but report the results from the full sample for completeness in the Supplementary Information.

**Table 2:** Multiple regression predicting each error with exGaussian parameters.

	$\beta$	*t*	*p*
**Dependent variable: Omission error**			
$\mu$	1.37	22.7	< 0.00001
$\sigma$	2.78	57.1	< 0.00001
$\tau$	3.63	60.2	< 0.00001
*R*^2^ = 0.398, *F*(3, 21,402) = 4710, *p* < 0.00001			
**Dependent variable: Commission error**			
$\mu$	− 2.44	− 79.1	< 0.00001
$\sigma$	2.04	81.9	< 0.00001
$\tau$	1.63	52.8	< 0.00001
*R*^2^ = 0.368, *F*(3, 21,402) = 4151, *p* < 0.00001

### Difference in exGaussian distribution parameters between brain states in Dataset2

**Figure 3:**
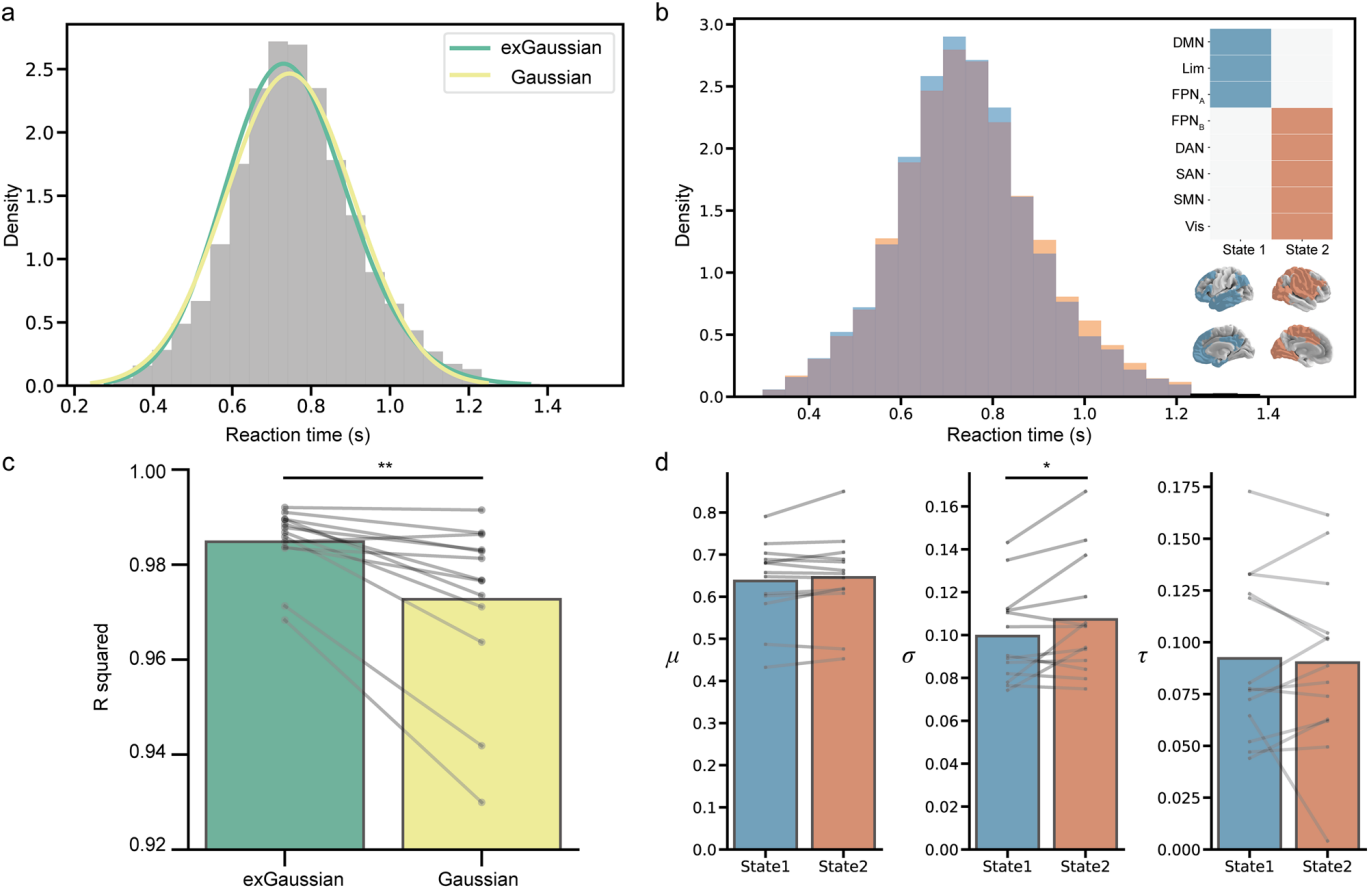
Results summary in Dataset2. (**a**) RT histogram and fitting result. (**b**) RT histograms in each brain state. Individual state is represented by an activity pattern in which each brain region is active (blue and red) or inactive (white). (**c**) R squared values for exGaussian and Gaussian distributions for each individual. (**d**) exGaussian parameters differences between brain states for each individual. *p < 0.05, **p < 0.005. DMN: default mode network; Lim: limbic; FPN: frontoparietal network; DAN: dorsal attention network; SAN: salience network; SMN: somatomotor network; Vis: visual.DMN: default mode network; Lim: limbic; FPN: frontoparietal network; DAN: dorsal attention network; SAN: salience network; SMN: somatomotor network; Vis: visual.

As we have shown in our previous studies^32^, we observed two dominant brain states during gradCPT using an energy landscape analysis (Fig3 b). State1 was characterized by higher activity in FPN_A_, DMN and limbic network, and was behaviorally optimal (high accuracy), while State2 was characterized by higher activity in FPN_B_, DAN, SN, SMN and visual network and was behaviorally suboptimal (low accuracy) (Supplementary Fig. S5a). This indicates that fluctuation of brain activity during gradCPT can be described as dynamic transitions between the two brain states represented by State1 and State2. In Dataset2, three of 16 participants had negative skewness and were excluded from the analysis. We first confirmed the exGaussian distribution better fit the data than the Gaussian distribution (*t*_12_ = 3.64, *p* < 0.005, Hedges’ *g* = 0.78, paired *t*-test, Fig. 3a,c). We then compared the exGaussian parameters between two brain states. We found only *σ* was significantly larger in State2 than State1 (*μ*: *t*_12_ = 1.35, *p* = 0.20, Hedges' *g* = 0.083; *σ*: *t*_12_ = 2.29, *p* < 0.05, Hedges' *g* = 0.31; *τ*: *t*_12_ = 0.32, *p* = 0.76, *g* = 0.048, paired *t*-test, Fig. 3b,d). This result indicates that the difference in RT variance between brain states was not due to difference in the long tail factor, but due to the difference in the variability factor. Note that when we estimated exGaussian parameters using flipped RT distribution for participants with negative skewness and included in analysis, the pattern of results did not change (Supplementary Fig. S2a).

### Replication using an independent dataset

**Figure 4:**
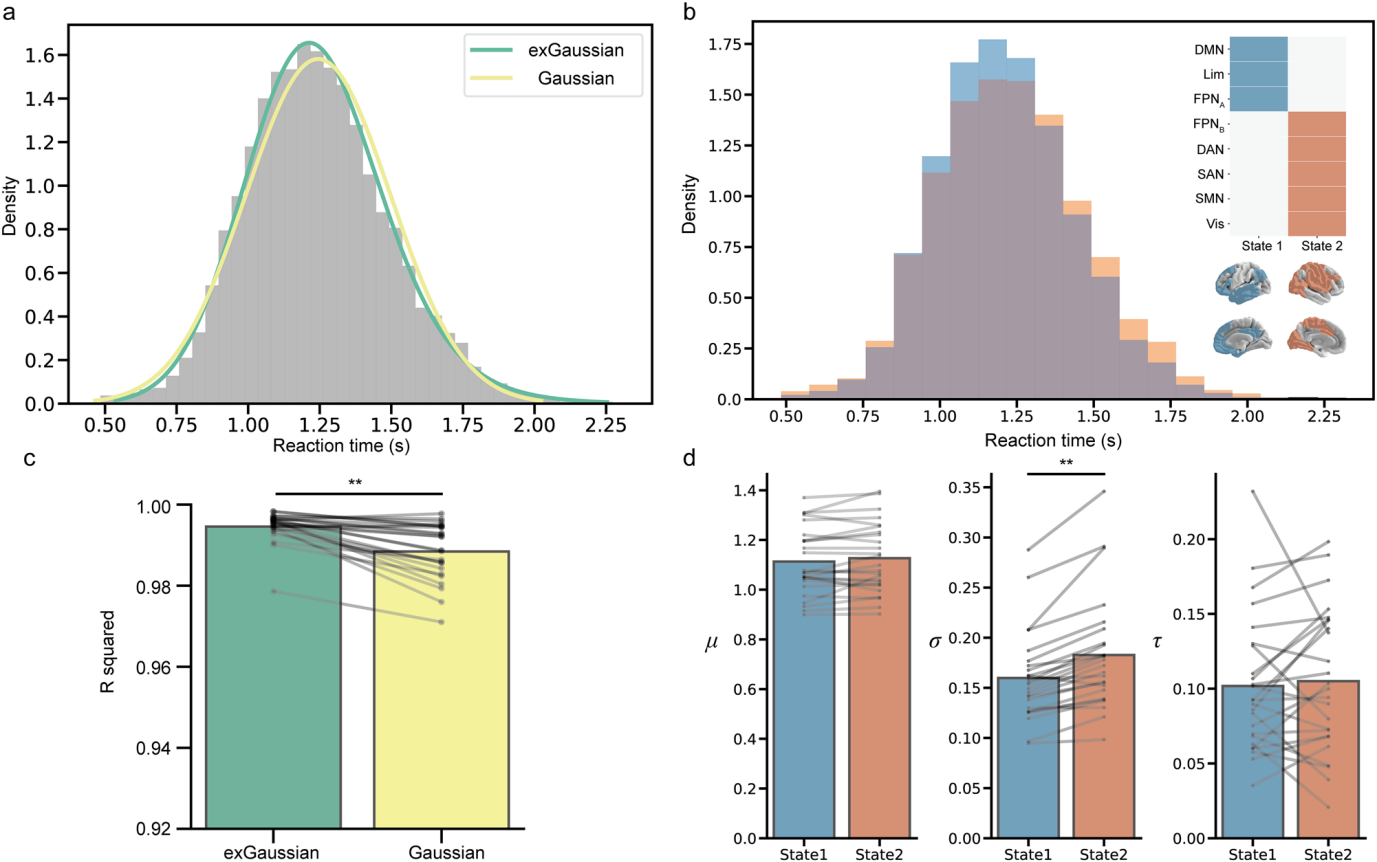
Results summary in Dataset3. (**a**) RT histogram and fitting result. (**b**) RT histograms in each brain state. Individual state is represented by an activity pattern in which each brain region is active (blue and red) or inactive (white). (**c**) R squared values for exGaussian and Gaussian distributions for each individual. (**d**) exGaussian parameters differences between brain states for each individual. **p < $1.0\times {10}^{-5}$. DMN: default mode network; Lim: limbic; FPN: frontoparietal network; DAN: dorsal attention network; SAN: salience network; SMN: somatomotor network; Vis: visual.DMN: default mode network; Lim: limbic; FPN: frontoparietal network; DAN: dorsal attention network; SAN: salience network; SMN: somatomotor network; Vis: visual.DMN: default mode network; Lim: limbic; FPN: frontoparietal network; DAN: dorsal attention network; SAN: salience network; SMN: somatomotor network; Vis: visual.DMN: default mode network; Lim: limbic; FPN: frontoparietal network; DAN: dorsal attention network; SAN: salience network; SMN: somatomotor network; Vis: visual.

We observed the same two dominant brain states in Dataset3 (Fig. 4b). State1 was characterized by higher activity in FPN_A_, DMN and limbic network, and was behaviorally optimal (high accuracy), while State2 was characterized by higher activity in FPN_B_, DAN, SN, SMN and visual network and was behaviorally suboptimal (low accuracy) (Supplementary Fig. S5b). In Dataset3, four of 29 participants had negative skewness and were excluded from analyses. We first confirmed that the exGaussian distribution better fit the data than the Gaussian distribution (*t*_24_ = 6.52, *p* < 0.001, Hedges’ *g* = 0.94, paired *t*-test, Fig. 4a,c). We then compared exGaussian parameters between two brain states. We found only *σ* was significantly larger in State2 than State1 (*μ*: *t*_24_ = 1.78, *p* = 0.089, Hedges' *g* = 0.099; *σ*: *t*_24_ = 6.29, *p* < 0.001, Hedges' *g* = 0.45; *τ*: *t*_24_ = 0.46, *p* = 0.65, Hedges' *g* = 0.069, paired *t*-test, Fig. 4b,d). Again, *σ* was higher in the optimal vs suboptimal brain state, which coincided with worse accuracy (Supplementary Fig. S5b). This result indicates that we successfully replicated our result in the independent dataset. Note that when we included estimated participants with negative skewness by estimating exGaussian parameters using flipped RT distribution, the pattern of results did not change (Supplementary Fig. S2b).

We further investigated the relationship between exGaussian parameters and mind wandering score across participants. We found a significant Spearman’s correlation coefficients between *σ* and mind wandering score (*μ*: *rho* = 0.17, *p* = 0.38; *σ*: *rho* = 0.56, *p* = 0.0018; *τ*: *rho* = 0.37 *p* = 0.0502, Supplementary Fig. S6). That is, the participants who tend to mind wandering had larger *σ*. Although we did not find significant correlation between τ and mind wandering score, statistical power may have been insufficient to find the relationship (*rho* = 0.37).

## Discussion

In the first part of this study, we aimed to disambiguate the ways in which reaction times are behavioral indicators of sustained attention. To do this, we verified the utility of exGaussian fitting to RT distributions in the gradCPT, and investigated the relationship between exGaussian parameters (*μ, σ*, and *τ*) and sustained attention performance (the number of omission error and commission error) using a large gradCPT dataset which included over 20,000 participants. We found that all exGaussian parameters were uniquely related to different aspects of sustained attention performance across individuals, with the variance factor most consistently related to all measures of accuracy. In the second part of this study, we tested the hypothesis that this variance factor represents different brain states (large variance hypothesis) as identified by an fMRI energy landscape analysis, vs. the alternative hypothesis that the long tail factor represents these different brain states (long tail hypothesis). Our results showed a significant difference in the variance factor (*σ)* between brain states, such that one brain state reflects optimal attention (lower variance and higher accuracy) and the other reflects suboptimal attention (higher variance and lower accuracy). We further replicated these results in a second independent dataset.

We found that *μ,* or the mean of the normal component of the reaction time, had moderate positive correlation with omission error and negative correlation with commission error. This result is consistent with previous reports that fast RTs are associated with commission error and slow RTs are associated with omission error^20^. That is, it is thought that *μ* represents either a strategy to reduce omission errors by speeding up the average RT or a strategy to reduce commission errors by slowing down the average RT^2,29,56^. This strategy factor, related to speed of reaction time, is also associated with a response bias to press (faster) or withhold (slower)^2^. We also found that *σ,* or the variance of the normal component of the reaction time, had moderate positive correlation with both omission error and commission error. We also found that *σ* was related to mind wandering. These results indicate that the variance factor is related to errors in general, regardless of the type of error. On the other hand, we found *τ,* or the exponential tail of the reaction time distribution, had moderate to large positive correlation with omission error but a smaller correlation with commission error. This result suggests that *τ* was strongly associated with occasional, more catastrophic attention lapses, or task disengagement^29,56^. These results indicate that fitting exGaussian could separate the strategy factor (*μ*), the variability factor (*σ*), and the long tail factor (*τ*), because all parameters have different relationships with sustained attention performance (the number of omission and commission errors). However, we note that interpretation of *τ* is difficult as we described in the “Methods” Section (“Estimation of exGaussian distribution parameters”), and therefore the interpretation should be treated with caution. Namely, in the gradCPT, this tail may manifest as both slow RTs (positive skew) or fast RT (negative skew), as slow responses to a given trial may be misassigned as fast responses to the next trial, given the gradual fading and some unavoidable ambiguity of a minority of responses (see “Methods”; Esterman et al. 2013)^21^. Similarly, given the response deadline implicit in this task, the long tail parameter may have been underestimated, and the reliability of this factor was far lower than the other RT factors, potentially reducing the ability to detect meaningful individual differences.

In our previous study^32^, we found across 4 independent datasets, that two dominant brain states, which were related to optimal and suboptimal attention, occurred during sustained attention. State1 was characterized by higher activity in FPN_A_, DMN and limbic network, and was behaviorally optimal, while State2 was characterized by higher activity in FPN_B_, DAN, SN, SMN and visual network and was behaviorally suboptimal. RT variance was lower and accuracy was higher during State1 than during State2, suggesting State1 was optimal and State2 was suboptimal. However, we did not previously analyze differences in RTs while accounting for their non-normality. In this study, we examined how RT distribution differed across two dominant brain states by comparing these exGaussian parameters characterized behaviorally in Dataset1. When doing so, we consistently found the variability factor (*σ*) was significantly different between brain states in two independent datasets. Note, although we did not find a significant difference in the long tail factor (*τ*) between brain states, it is possible that *τ* was not different due to the nature of the experimental task. That is, since the gradCPT has a short ISI (800 or 1300 ms), extremely long RTs were not possible. This may also have contributed to the relatively lower reliability of this factor. Despite this limitation, these results suggests that both slow and fast RTs are induced by same suboptimal brain state (State2) characterized as activation of DAN, SAN, SMN, FPN_B_ and visual networks.

There are a number of potential interpretations of the variance factor. A previous study suggested that variability may represent exploration from the viewpoint of exploring (variable) and exploiting (stable) trade-offs^33^. For example, participants with large variability exhibit faster learning than participants with small variability^57^. We speculate that State1 may be the exploitative state and State2 may be the explorative state, with regard to response strategy or approach to the task. For example, when accuracy is higher, participants may continue to use the same approach, leading to more consistent RTs, whereas when performance is worse, participants may explore different approaches, leading to more variable RTs (both faster and slow). Alternatively, it could be that greater RT variability reflects greater noise in the perceptual, selection, or response system. For example, Rothlein et al. (2018) found that during more variable periods of task performance, representational fidelity of stimulus representations was weaker in the visual cortices^58^. A further related possibility is that suboptimal states represent less automatic processing of the stimuli alongside higher perceptual load^21,55^, which may lead to greater complexity of behavior reflected in higher variability.

While variability during sustained attention is likely to fluctuate due to intrinsic (task-unrelated) factors, there are a number of ways in which extrinsic factors can impact response variability. Our previous studies show that variability is reduced in conditions where participants are rewarded based on their performance^55,59,60^. Furthermore, motor variability is actively regulated by recent reward history, with variability increasing when performance is poor^33,61^. On the other hand, our previous study found reactive DAN/SN activation in response to errors^60,62^. This suggests that the more variable and suboptimal brain state (State2) also may be partly driven by response to errors. Together, this kind of reward (error)-dependent regulation of variability can be interpreted as an exploitation-exploration dilemma. In other words, while the reward is being obtained, variance is reduced and the current action (or strategy) is continued; when the reward is not obtained (or the participant makes an error), variance increases and another action is taken to change the current action. There is no consensus whether or how variability is regulated in the brain^63,64^. Further study is needed to investigate when brain state transition occurs, as well how variability changes in relationship to extrinsic factors.

As a limitation of our current study, although we did not find a significant difference in the long tail factor (*τ*) between brain states, our results do not rule out the possibility that fast and slow RTs are induced from different brain mechanisms (fast RTs represent mindless and automatic processing, and slow RTs represent disengaged/inefficient processing). This is because it is possible that *τ* was not different due to the nature of the gradCPT as we discussed above. As such, these results may not generalize beyond the gradCPT, or other CPTs with short response deadlines. The similarities and differences in cognitive mechanisms between gradCPT and other sustained attention task such as PVT, which do not have a response deadline, are important and interesting research topics. Nonetheless, given the ubiquity of the gradCPT and similar task in the field^2,4,14^, the current results are relevant to the field. Critically, our results provide evidence of the existence of a stable RT brain state and a variable RT brain state independent of a long tail during this type of sustained attention task. Another limitation is that our results do not address any causal relationship between brain state and behavior. Some evidence suggests, from our previous studies, that activation of DAN and salience network, which are included in suboptimal State2, occur in response to omission and commission errors^26,60^. Therefore, the State2-related networks may be driven by reactivity to errors or increased variability. Similarly, DMN activity is associated with the optimal state (State1) and low variability, but also higher degrees of mind wandering during gradCPT^35^, suggesting a complex role of DMN in sustained attention. Therefore, further study is needed to clarify the causal relationships between network activity and sustained attention. This may be possible by using real time presentation of stimuli according to brain state^65^. Additionally, event-related TMS of these networks could further help infer causality^66,67^.

In summary, we revealed all parameters of the exGaussian were uniquely related to sustained attention performance based on data from a large sample. *μ* is the strategy factor, *σ* is the variability factor, and *τ* is the long tail factor, respectively. Our results showed the utility of the exGaussian distribution for relating RTs to sustained attention. We further revealed that the variance factor is significantly different between two dominant brain states during a sustained attention task. This result suggests that the suboptimal brain State2, relative to the optimal brain State1, is not characterized by slower RTs, but rather by increased variance of RTs (and reduced accuracy). We believe that our findings better identify and refine a behavioral marker of optimal sustained attention based on brain states, and represent an important theoretical and methodological finding for future sustained attention research.

## Supplementary Information

Supplementary Information.

## Data Availability

Analysis code and summary data required to reproduce all figures in our manuscript is publically available (https://github.com/Ayumu722/ScientificReports_exGaussian). However, a portion of the raw data (e.g. brain images) are owned by the United States Department of Vet- erans Affairs and are available only upon request from the United States Department of Veterans Affairs. The Department of Veterans Af- fairs will make this data publicly available and requests for the data can be made by interested individuals by filing a Freedom of Infor- mation Act request to the Privacy Officer at VA Boston Healthcare System (vhabhsFOIAofficers@va. gov) or the FOIA Intake Center (see http://www.oprm.va.gov/foia/foia_howTo.aspx for more details).
