# Supplementary material for: Variable rather than extreme slow reaction times distinguish brain states during sustained attention
Source: Sci Rep. 2021 Jul 21;11:14883. doi: 10.1038/s41598-021-94161-0 (PMC8295386; doi:10.1038/s41598-021-94161-0)
Supplement: Supplementary file 1 — Supplementary Information. [file 41598_2021_94161_MOESM1_ESM.docx]

**Supplementary Materials**


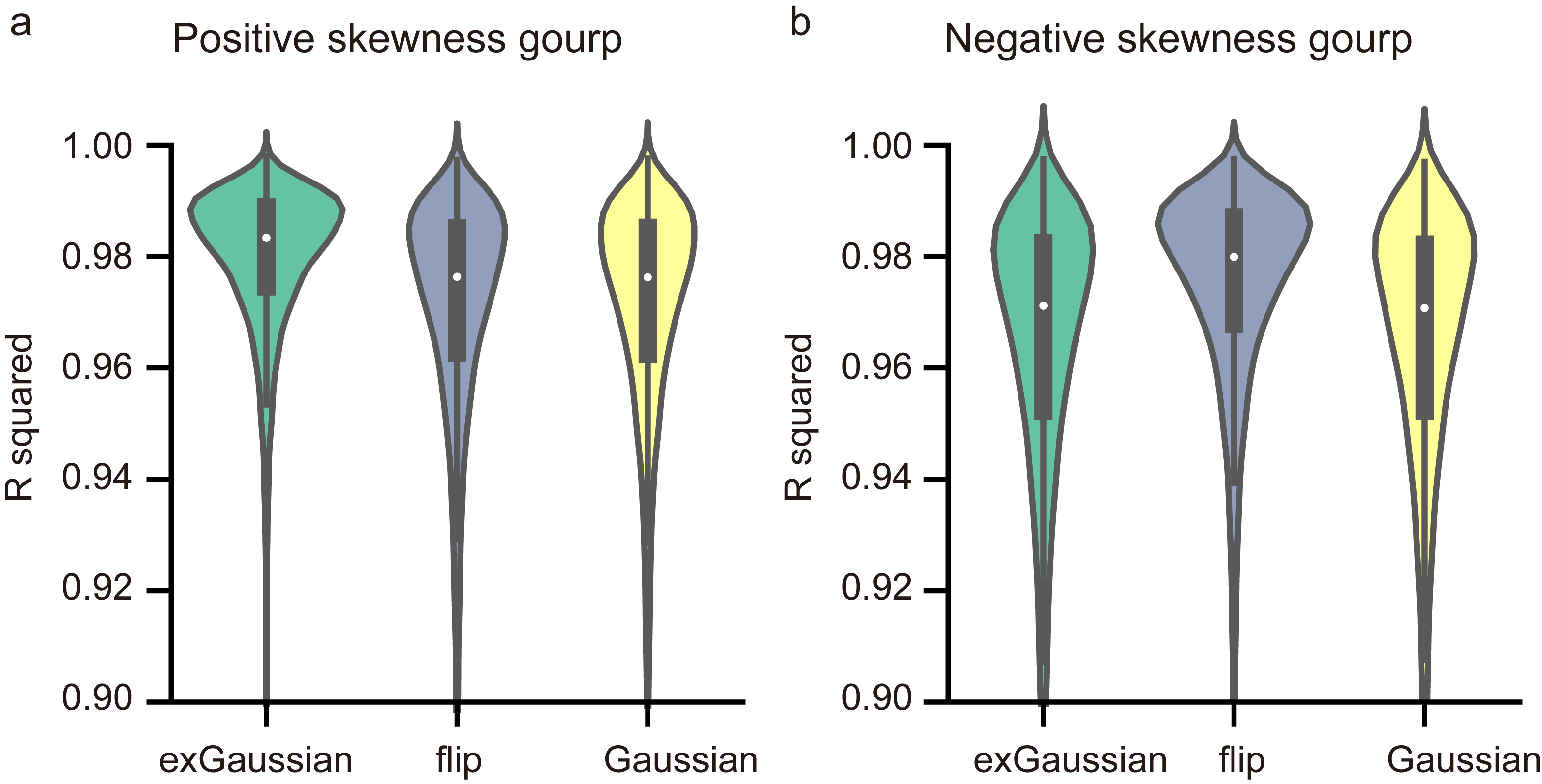


**Supplementary Figure 1. Goodness of fit in Dataset1.** (a) R squared values of exGaussian, flipped exGaussian, Gaussian distributions in the positive skewness group, respectively. (b) R squared values of exGaussian, flipped exGaussian, Gaussian distributions in the negative skewness group, respectively.


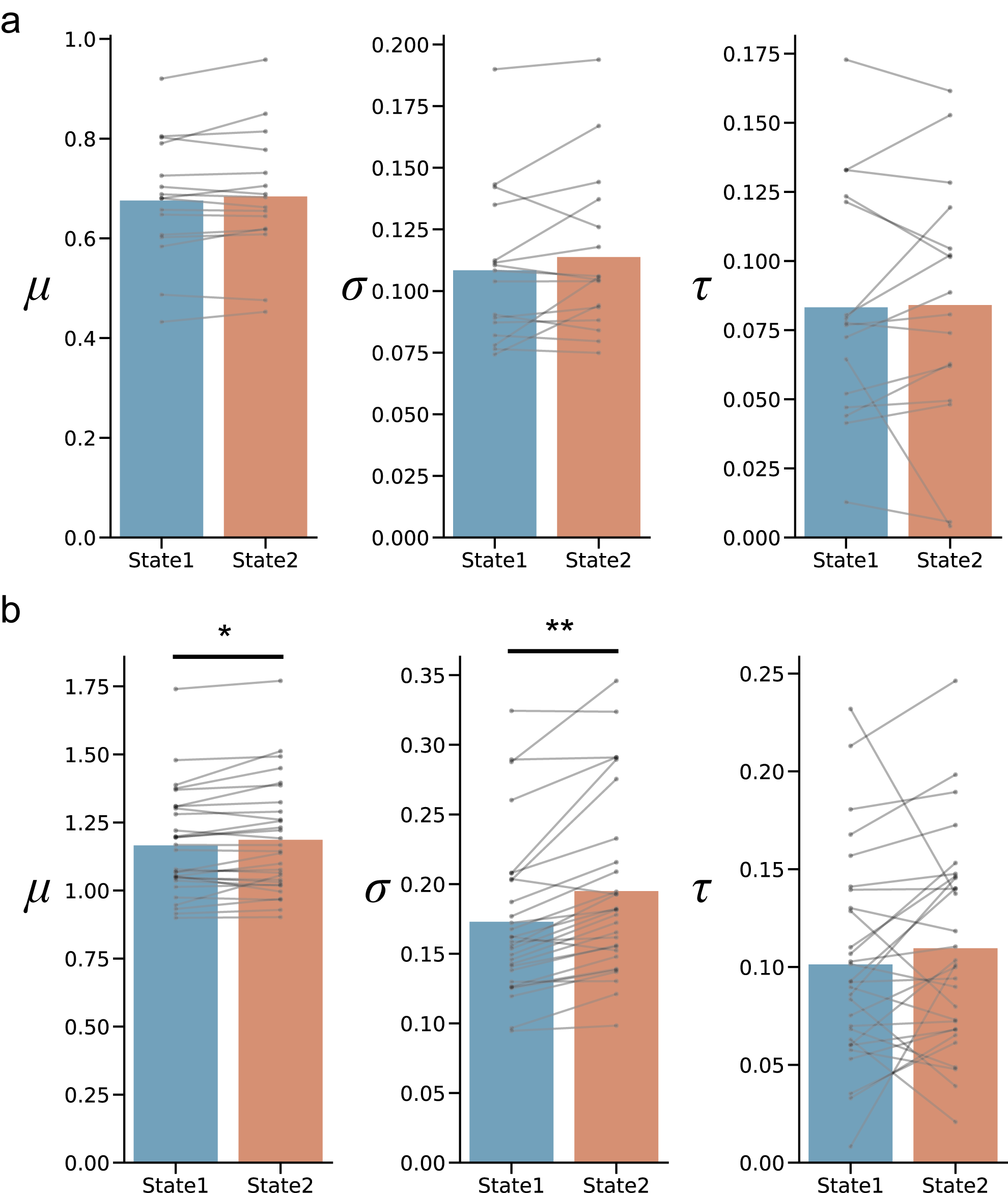


**Supplementary Figure 2. exGaussian parameters differences between brain states including individual with negative skewness.** (a) Results in Dataset2. (b) Results in Dataset3. * *p* < 0.05, ** *p* < $1.0\times{10}^{-5}$.


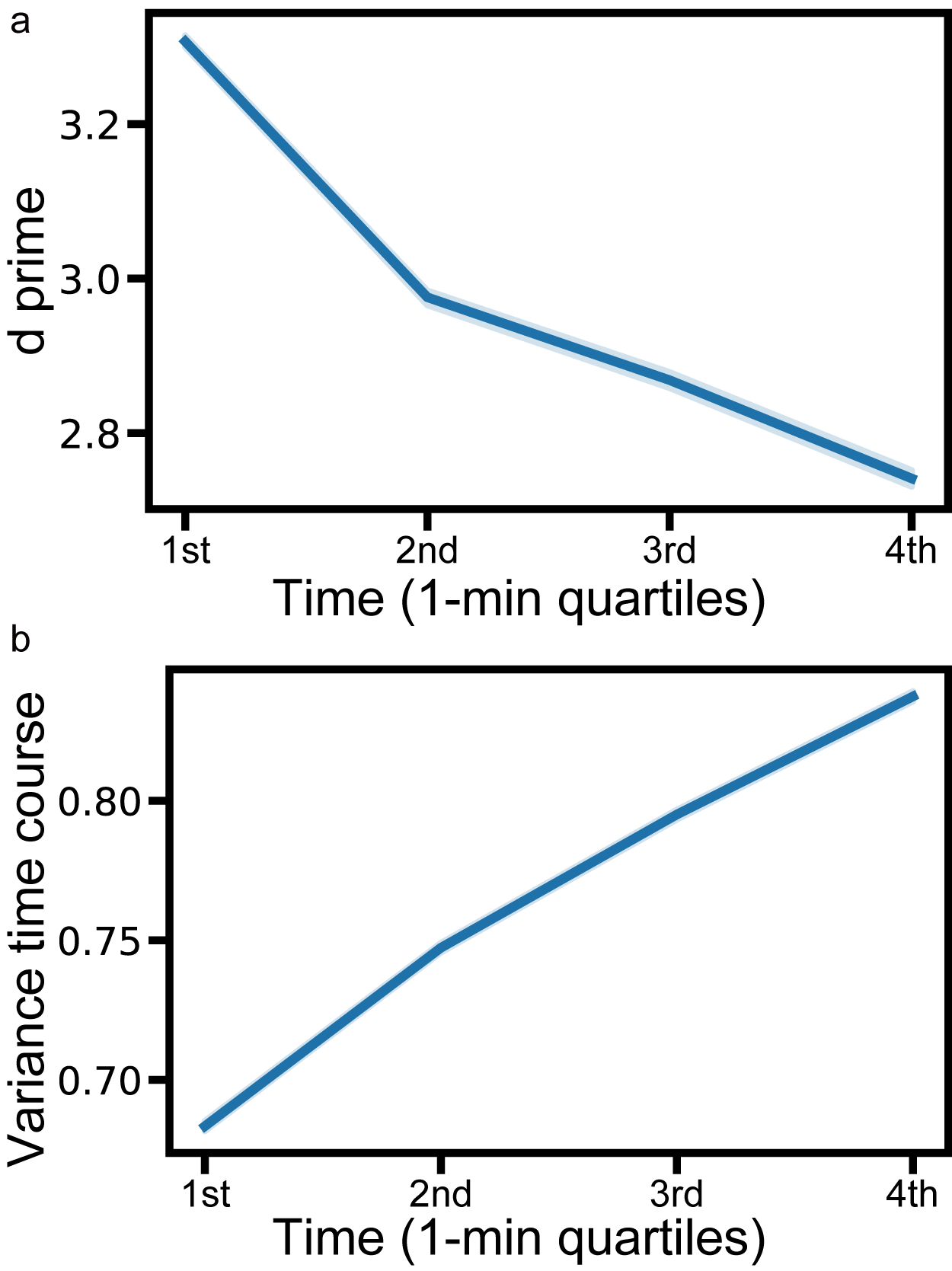
**Supplementary Figure 3. Performance decrement over time in Dataset1.** (a) dprime decreased over time, across the 4 1-minute quartiles (slope: *β* = -0.18, *p* < 0.0001; intercept: *β* = 0.34, *p* < 0.0001, linear regression analysis *t*-test). Solid line indicates mean across participants and shaded area reflects 95% confidence interval of the mean. d prime is an index of accuracy, or perceptual sensitivity, that can simultaneously represent hit rate and false alarm rate. In this case, a hit indicates correct omission to the mountain image (correct omission), and a false alarm is a failure to response to a city scene (omission error); d prime was calculated as z(hit rate) − z(false alarm rate). Here z is normal probability density. (b) Variance time course (VTC) increased over time (slope: *β* = 0.05, *p* < 0.0001; intercept: *β* = 0.64, *p* < 0.0001, linear regression analysis *t*-test). Solid line indicates mean across participants and shaded area reflects 95% confidence interval of the mean. VTCs were computed from the correct responses in each participant (following z-transformation of RTs within-subject to normalize the scale of the VTC), where the value assigned to each trial represented the absolute deviation of the trial’s RT from the mean RT of the run.


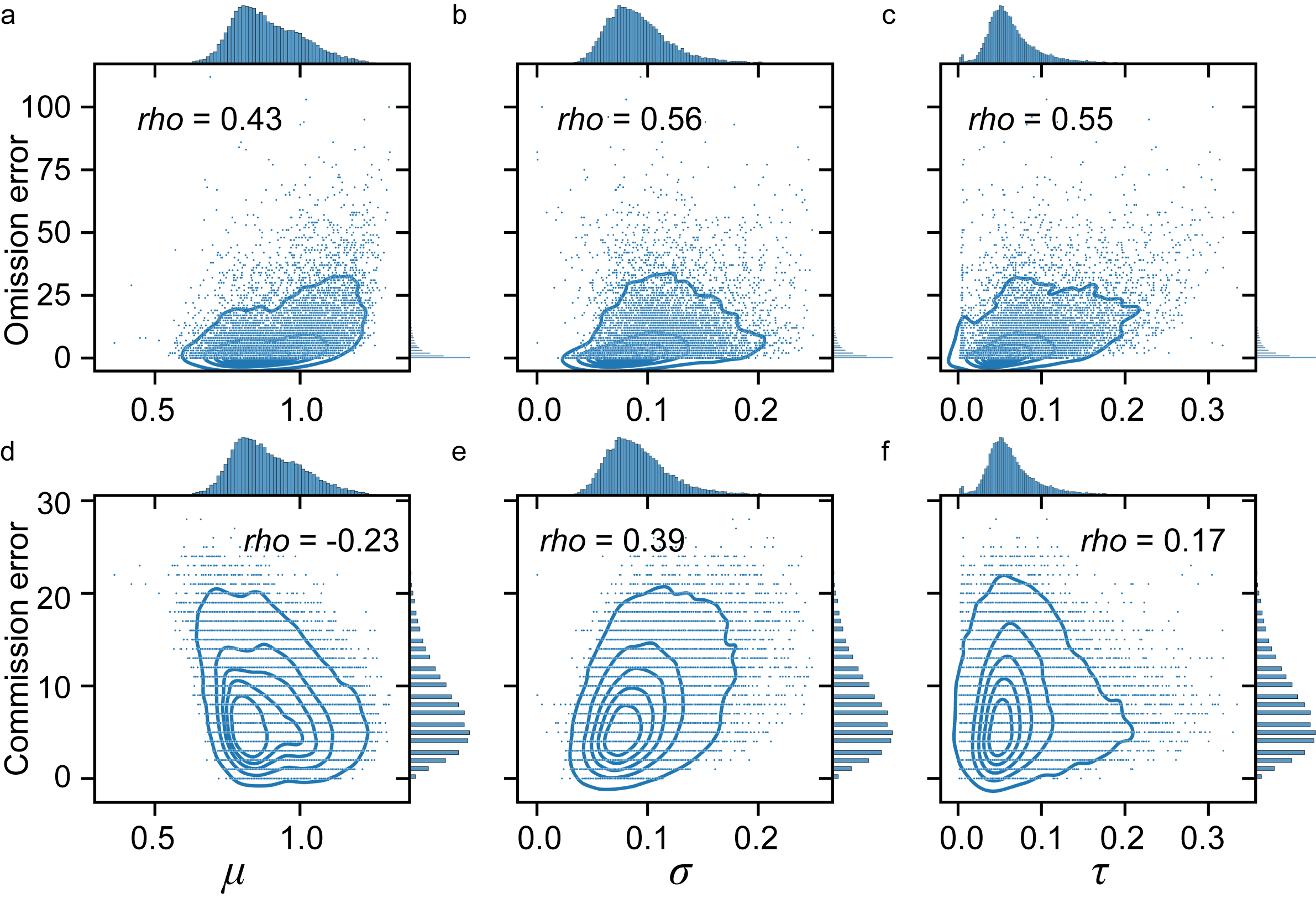
**Supplementary Figure 4. Relationship between exGaussian distribution parameters and sustained attention performances in whole group.** (a) Scatter plot and histograms of *μ* and the number of omission errors. (b) Scatter plot and histograms of *σ* and the number of omission errors. (c) Scatter plot and histograms of *τ* and the number of omission errors. (d) Scatter plot and histograms of *μ* and the number of commission errors. (e) Scatter plot and histograms of of *σ* and the number of commission errors. (f) Scatter plot and histograms of *τ* and the number of commission errors. Solid line indicates a kernel density estimate, which is a method for visualizing the distribution of observations. Spearman’s correlation coefficients values were shown in each panel.


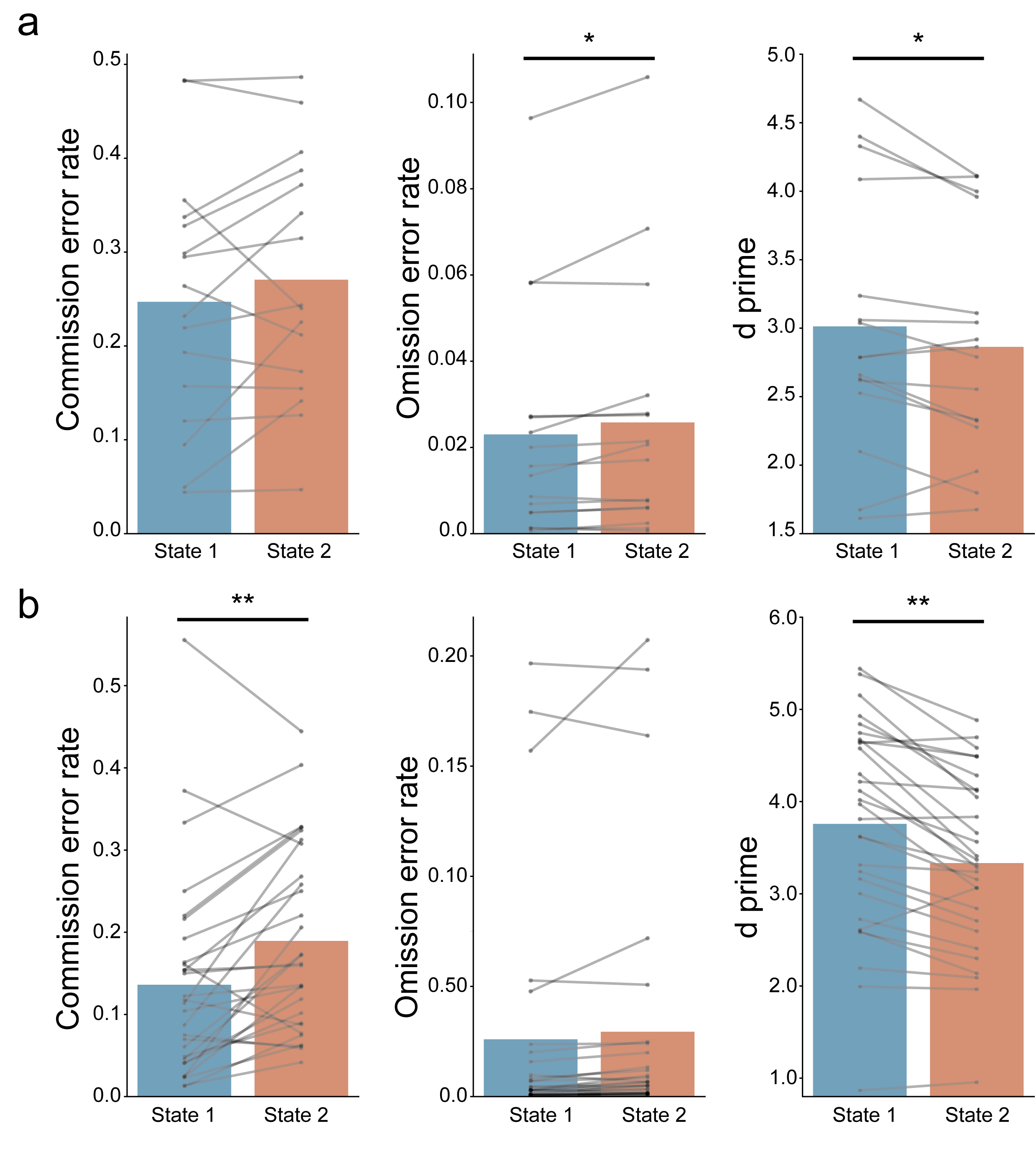


**Supplementary Figure 5. Accuracy differences between brain states.** (a) Results in Dataset2. (b) Results in Dataset3. * *p* < 0.05, ** *p* < $1.0\times{10}^{-4}$. d prime is an index of accuracy, or perceptual sensitivity, that can simultaneously represent hit rate and false alarm rate. In this case, a hit indicates correct omission to the mountain image (correct omission), and a false alarm is a failure to response to a city scene (omission error); d prime was calculated as z(hit rate) − z(false alarm rate). Here z is normal probability density.


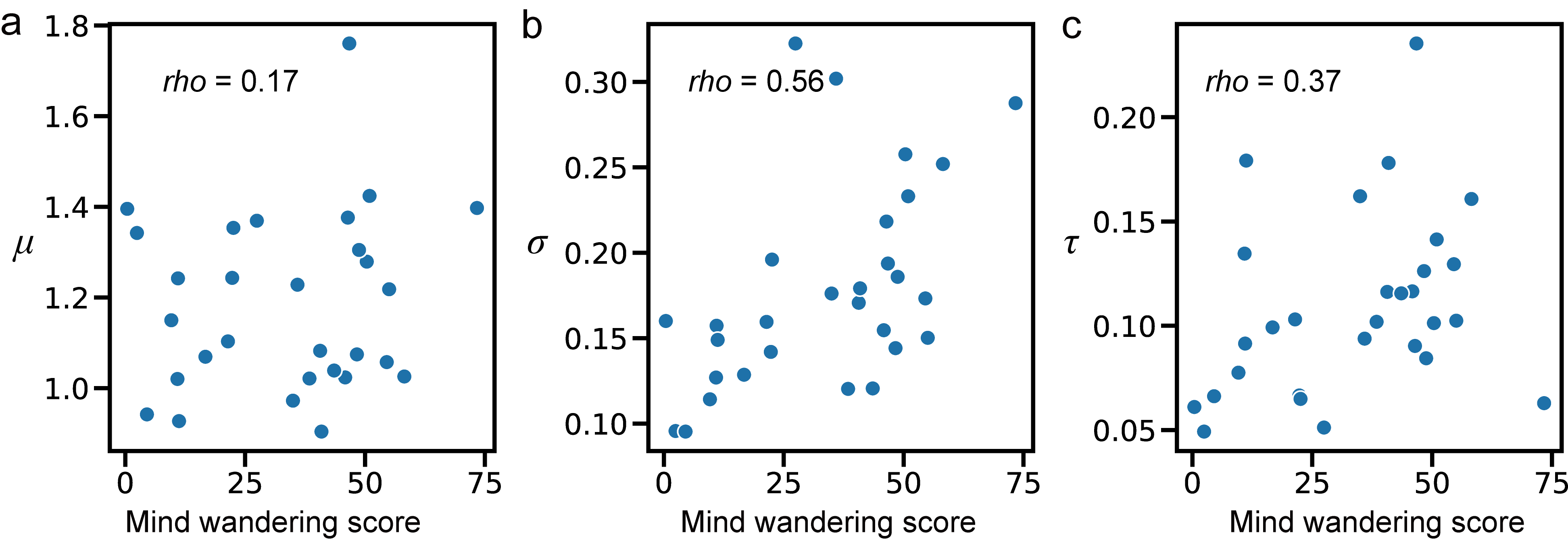


**Supplementary Figure 6. Relationships between mind wandering score and exGaussian parameters.** (a) Scatter plot between mind wandering score and *μ*. (b) Scatter plot between mind wandering score and *σ*. (c) Scatter plot between mind wandering score and *τ*. Pearson’s correlation coefficients values were shown in each panel.
